# Fire blight QTL analysis in a multi-family apple population identifies a reduced-susceptibility allele in ‘Honeycrisp’

**DOI:** 10.1038/s41438-021-00466-6

**Published:** 2021-02-01

**Authors:** Sarah A. Kostick, Soon Li Teh, John L. Norelli, Stijn Vanderzande, Cameron Peace, Kate M. Evans

**Affiliations:** 1 Tree Fruit Research and Extension Center, Department of Horticulture, Washington State University, Wenatchee, WA 98801 USA; 2 United States Department of Agriculture, Agricultural Research Service, Appalachian Fruit Research Laboratory, Kearneysville, WV 25430 USA; 3 Department of Horticulture, Washington State University, Pullman, WA 99164 USA

**Keywords:** Agricultural genetics, Plant breeding, Plant genetics

## Abstract

Breeding apple cultivars with resistance offers a potential solution to fire blight, a damaging bacterial disease caused by *Erwinia amylovora*. Most resistance alleles at quantitative trait loci (QTLs) were previously characterized in diverse *Malus* germplasm with poor fruit quality, which reduces breeding utility. This study utilized a pedigree-based QTL analysis approach to elucidate the genetic basis of resistance/susceptibility to fire blight from multiple genetic sources in germplasm relevant to U.S. apple breeding programs. Twenty-seven important breeding parents (IBPs) were represented by 314 offspring from 32 full-sib families, with ‘Honeycrisp’ being the most highly represented IBP. Analyzing resistance/susceptibility data from a two-year replicated field inoculation study and previously curated genome-wide single nucleotide polymorphism data, QTLs were consistently mapped on chromosomes (Chrs.) 6, 7, and 15. These QTLs together explained ~28% of phenotypic variation. The Chr. 6 and Chr. 15 QTLs colocalized with previously reported QTLs, while the Chr. 7 QTL is possibly novel. ‘Honeycrisp’ inherited a rare reduced-susceptibility allele at the Chr. 6 QTL from its grandparent ‘Frostbite’. The highly resistant IBP ‘Enterprise’ had at least one putative reduced-susceptibility allele at all three QTLs. In general, lower susceptibility was observed for individuals with higher numbers of reduced-susceptibility alleles across QTLs. This study highlighted QTL mapping and allele characterization of resistance/susceptibility to fire blight in complex pedigree-connected apple breeding germplasm. Knowledge gained will enable more informed parental selection and development of trait-predictive DNA tests for pyramiding favorable alleles and selection of superior apple cultivars with resistance to fire blight.

## Introduction

The development of apple (*Malus domestica* Borkh.) cultivars with genetic resistance offer a potentially sustainable solution to fire blight, a damaging bacterial disease caused by *Erwinia amylovora*. This pathogen can cause severe structural tree damage and death, resulting in substantial economic losses^1^. Apple production systems have become more vulnerable to fire blight due to widespread planting of susceptible apple cultivars, adoption of high-density planting systems, and lack of control methods effective against all disease phases^1^. Resistance to fire blight is an important goal of many apple breeding programs^2,3^.

Phenotypic evaluation of the degree of resistance or susceptibility to fire blight can be difficult due to quantitative host resistance, differential host × *E. amylovora* strain interactions, strong environmental influences on disease incidence, and effects of tree vigor on susceptibility^4–6^. Segregation for resistance/susceptibility has been observed in susceptible × susceptible families^7^. As a result, phenotypic information alone is not predictive of performance and thus is inadequate to guide breeding decisions targeting resistance to fire blight.

Several quantitative trait loci (QTLs) additive and/or epistatic associated with resistance/susceptibility to fire blight have been detected throughout the apple genome^8–20^. Multiple QTLs have been mapped in wild *Malus* germplasm (e.g., ‘Evereste’, *M. floribunda* 821, *M. robusta* 5) characterized by astringent, crabapple-type fruit, with non-immediate breeding utility for apple scion improvement^8^. Introgression of resistance alleles from wild sources is possible; however, improving fruit quality while maintaining resistance is challenging due to long generation times, gametophytic self-incompatibility, and high heterozygosity of *Malus* germplasm^4,21,22^. QTLs on chromosomes (Chrs.) 2, 3, 5, 6, 7, 8, 9, 10, 13, and 15 have been mapped in populations derived from cultivars with most of these QTLs accounting for ≤ 20% of phenotypic variation^9,15–17,20^. Of such QTLs detected in elite backgrounds, only one has been validated in multiple studies, located on Chr. 7, with the low-susceptibility allele originating from the cultivar Cox’s Orange Pippin, and explaining ~30–50% of phenotypic variation^9,15,20^. Several other cultivars including Aurora Golden Gala, Delicious, Empire, and Frostbite have demonstrated low relative susceptibility^23^; however, little is known about the genetic basis of resistance of these cultivars. Resistance to fire blight is therefore an attractive target for DNA-informed breeding (e.g., marker-assisted parent, marker-assisted seedling selection). However, apple breeders are currently limited by the few trait-predictive DNA tests that are available (summarized by Evans and Peace^24^).

Current understanding of the genetic basis of resistance/susceptibility to fire blight of germplasm relevant to U.S. apple breeding programs is insufficient for the immediate development of DNA tests. Relevant sources and effects of QTL alleles need to be determined prior to the development and deployment of trait-predictive DNA tests^25^. Pedigree-based QTL analysis (PBA)^26,27^ enables the integrated analysis of multiple small pedigree-connected full-sib families. As populations of such germplasm are typical of populations routinely generated and evaluated in breeding programs, detected QTLs are simultaneously validated for breeding relevance^27^. PBA has several further advantages compared to biparental QTL studies, including increased statistical power, representation of wider genetic diversity, and the ability to determine ancestral sources of QTL alleles^27,28^. PBA has been successfully applied in apple for numerous traits^26,29–34^, including resistance/susceptibility to fire blight^20^. Key experimental resources are available to characterize genetic factors that influence complex traits in apple, including representative pedigree-connected germplasm sets^28^, high-quality genome-wide single nucleotide polymorphism (SNP) data^35,36^, and FlexQTL^TM^ software (www.flexqtl.nl)^26,37–39^.

This study aimed to elucidate the genetic basis of resistance/susceptibility to fire blight in a large set of pedigree-connected germplasm relevant to U.S. apple breeding programs. The objectives were to (1) detect QTLs associated with resistance/susceptibility to fire blight; (2) characterize effects of alleles at stable QTLs represented by multi-SNP haplotypes; and (3) determine frequencies of putative reduced- and increased-susceptibility alleles among apple cultivars, important breeding parents (IBPs), and progenitors. It was hypothesized that multiple QTLs associated with resistance/susceptibility to fire blight would be detected.

## Results

### QTL detection

In both years, QTLs were detected on Chrs. 6, 7, and 15 with positive (Bayes Factor; BF; 2lnBF_10_ > 2) to strong (BF > 5) evidence (Table 1 and Supplementary Table S1; Supplementary Figs. S1, S2, S3, and S4). QTL modes varied by less than 5 cM across years, with ranges of 49–53 cM, 24–28 cM, and 68–72 cM for the Chr. 6, 7, and 15 QTLs, respectively (Table 1 and Supplementary Table S1). There was positive evidence for a second QTL on Chr. 7 in 2016 (located at 52–66 cM, 28.98–33.23 Mbp) but not in 2017. In both years, there was strong to decisive (BF > 10) evidence for at least one QTL on Chr. 8 and positive evidence for a second Chr. 8 QTL (Table 1 and Supplementary Table S1). The two possible Chr. 8 QTL intervals in 2017 were 22–40 cM (9.4–22.0 Mbp) and 52–64 cM (27.5–30.9 Mbp). In 2016, there was strong evidence for a QTL on Chr. 16 but no evidence in 2017 (Table 1). There was no evidence for any QTLs in the rest of the genome in either year (data not shown). Use of adjusted SLB BLUPs estimated across years as phenotypic values did not provide further insights as results were similar to results of QTL analyses with within-year adjusted SLB BLUPs as phenotypic values (results not presented). A dominance model did not provide further insights as there was no evidence for dominance effects or additional QTLs (data not presented).

**Table 1:** Summary of resistance/susceptibility to fire blight QTL analyses in the pedigree-connected apple reference germplasm set representing 27 important breeding parents (IBPs)

Chr.^a^	Year	BF_(1 vs. 0)_^b^	BF_(2 vs. 1)_^c^	QTL interval (cM)^d^	Mode (cM)^e^	Physical position (Mbp)^f^	PVE (%)^g^
6	2016	11.5	0.7	48–58	53	31.22–36.72	15
	2017	4.8	−0.2	42–56	49	28.24–35.97	9
7	2016	7.2	2.1	22–32	24	9.11–20.90	10
	2017	4.0	0.3	22–32	28	9.11–20.90	9
8	2016	5.4	0.9	40–64	52	21.97–30.88	–
	2017	15.4	3.0	22–40	27	9.42–21.97	–
15	2016	3.8	0.5	56–76	68	19.23–30.29	6
	2017	5.7	0.7	58–82	72	20.09–37.66	7
16	2016	5.2	1.7	0–12	9	0.02–5.14	–
	2017	−0.5	−0.7	–	–	–	–

Within a year, adjusted shoot length blighted best linear unbiased predictions (SLB BLUPs) were used as phenotypic values in QTL analyses. Results are shown for a single representative FlexQTL^TM^ run within each year (all FlexQTL^TM^ runs presented in Table S1)

^a^Chromosome

^b^Chromosome-wise Bayes factor (2lnBF_10_) for a 1 QTL vs. 0 QTL model, with BF > 2, 5, and 10 indicating positive, strong, or decisive evidence, respectively, for the presence of one QTL

^c^Chromosome-wise Bayes factor (2lnBF_10_) for a 2 QTL vs. 1 QTL model, with BF > 2, 5, and 10 indicating positive, strong, or decisive evidence, respectively, for the presence of two QTLs

^d^QTL interval defined as the bounds of consecutive 2 cM bins (chromosomal segments used by and reported from FlexQTL™) with 2lnBF_10_ (BF) > 2

^e^Mode of QTL interval, representing the most probable QTL position

^f^Approximate physical position of QTL interval, from physical positions on GDDH13 v1.1 reference genome (Daccord et al.^21^) of closest flanking SNPs according to Vanderzande et al.^36^

^g^Estimated proportion of phenotypic variance explained by QTL

### Functional annotations of genes in chromosomes 6, 7, and 15 QTL intervals

The physical position of the Chr. 6 QTL overlapping interval between results of 2016 and 2017 spanned 4.8-Mbp (31.2–36.0 Mbp; Table 1) and contained 423 functionally annotated genes, including 74 (18%) genes putatively involved in responses to diseases and biotic stresses (Supplementary Table S2). Of the 369 annotated genes in the common 11.7-Mbp QTL interval on Chr. 7, annotations for 81 genes (22%) indicated involvement in responses to diseases and biotic stresses (Table S2). The physical position of the Chr. 15 QTL overlapping interval spanned 10.2-Mbp (20.1–30.3 Mbp; Table 1) with 407 annotated genes, 90 (22%) of which have putative roles in responses to diseases and biotic stresses (Supplementary Table S2).

### FlexQTL^TM^ assignment of QTL genotypes for IBPs

Some IBPs were successfully assigned estimates of QTL genotypes at each of the three stable QTLs (Chrs. 6, 7, and 15) under a bi-allelic model, where *q* was associated with relatively low adjusted SLB BLUPs (i.e., lower susceptibility), compared to the higher-susceptibility *Q* allele. At the Chr. 6 QTL, several IBPs (‘Aurora Golden Gala’, ‘Enterprise’, ‘Golden Delicious’, ‘Pinova’, ‘WA 5’, and W.7) were estimated to be homozygous *qq*, while ‘Honeycrisp’ was estimated to be heterozygous (*Qq*) and no IBPs were *QQ* (Supplementary Table S3). At the Chr. 7 QTL, multiple IBPs (‘Arlet’, BC 8S-27–43, ‘Cripps Pink’, ‘Enterprise’, ‘Fuji’, ‘Splendour’, and ‘WA 5’) were assigned as *qq*, ‘Golden Delicious’ and ‘Pinova’ as *Qq*, and ‘Sweet Sixteen’ as *QQ* (Supplementary Table S3). Several IBPs (‘Delicious’, ‘Enterprise’, ‘Granny Smith’, ‘Minnewashta’, ‘Sansa’) were *qq* at the Chr. 15 QTL, while ‘Aurora Golden Gala’, BC 8S-27-43, ‘Cripps Pink’, ‘Splendour’, and ‘WA 5’ were estimated to be *Qq* (Supplementary Table S3). ‘Pinova’ and ‘Silken’ were estimated to be *QQ* at the Chr. 15 QTL (Supplementary Table S3). However, QTL genotype assignments for other IBPs by FlexQTL^TM^ software were often inconclusive and some IBP QTL genotype estimates were inconsistent across years (Supplementary Table S3).

### Chromosome 6 QTL haplotype analysis

**Fig. 1:**
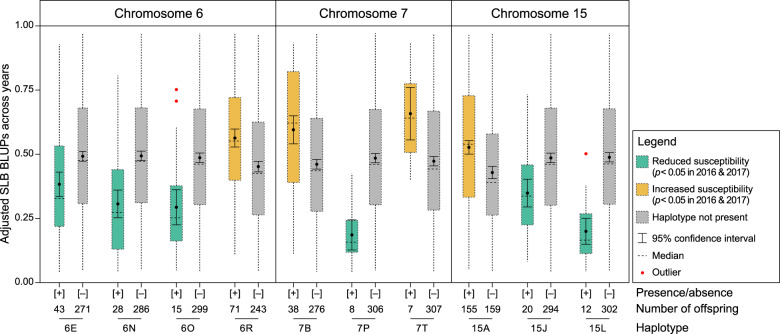
Distributions, means, and 95% confidence intervals of adjusted shoot length blighted best linear unbiased predictions (SLB BLUPs) across years for 314 apple offspring with and without significant haplotypes at QTLs on chromosomes 6, 7, and 15. For each haplotype, an ANOVA was used to determine whether the presence of the haplotype had a significant effect (*p* < 0.05) within a year

Eighteen IBP Chr. 6 QTL haplotypes, constructed from 25 SNPs spanning 5.7 cM (2.3-Mbp; Supplementary Table S4), segregated in the full-sib families (Supplementary Table S5). Four Chr. 6 QTL haplotypes (6E, 6N, 6O, 6R) exhibited significant effects in both 2016 and 2017 (*p* < 0.05; Fig. 1). Three further Chr. 6 QTL haplotypes (6C, 6G, 6M) had significant effects in 2016 (*p* < 0.05) but not 2017 (*p* > 0.05). The other 11 Chr. 6 QTL haplotypes were not associated with significant effects in either year (*p* > 0.05). In general, offspring that inherited 6E (*n* = 43), 6N (*n* = 28), or 6O (*n* = 15) demonstrated low to moderate susceptibility responses, with adjusted SLB BLUP means across years of 0.38, 0.30, and 0.30, respectively (Fig. 1). An offspring that had both 6E and 6N demonstrated the lowest adjusted SLB BLUPs of all phenotyped individuals in both years (Supplementary Table S6). In contrast, offspring with the ‘Honeycrisp’-derived 6R haplotype exhibited increased-susceptibility responses with an adjusted SLB BLUP mean of 0.56 (Fig. 1).

**Fig. 2:**
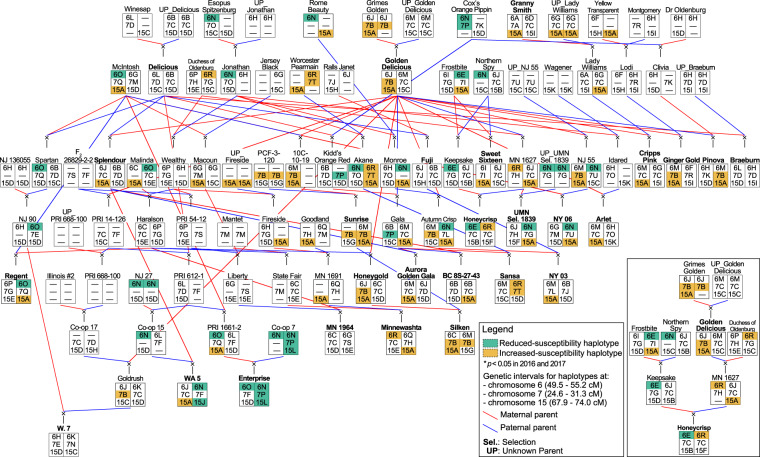
Pedigree relationships among 27 important breeding parents (in bold) represented in the subset of the U.S. Apple Crop Reference and Breeding Pedigree Sets. Chromosome 6, 7, and 15 QTL haplotypes were traced through pedigrees to furthest ancestors. Honeycrisp’s pedigree was repeated as an inset figure to clearly depict inheritance of reduced-susceptibility haplotype 6E and increased-susceptibility haplotype 6R. For a given individual, haplotype listing order was determined based on phasing information. Unknown parents are designated by UP_xxx. “–” refers to other progenitor haplotypes not segregating in full-sib families or unresolved haplotypes. Colorized haplotypes were those with significant effects (*p* < 0.05) in both 2016 and 2017. For better visibility of complex lineages, uneven vertical scaling between pedigree rows was used

The reduced-susceptibility haplotype of ‘Honeycrisp’, 6E, was traced to ‘Frostbite’ (Fig. 2 and Supplementary Table S5). The reduced-susceptibility 6N haplotype of IBPs ‘Enterprise’, NY 06, UMN selection 1839, and ‘WA 5’ was traced to multiple ancestors: Co-op 7, NJ 55, NJ 27, and unknown parent of UMN selection 1839 (Fig. 2 and Supplementary Table S5). Haplotype 6N in ‘Enterprise’ was identical-by-state with several cultivars including ‘Cox’s Orange Pippin’ (Fig. 2). ‘Enterprise’ and ‘Regent’ each had one copy of reduced-susceptibility haplotype 6O that traced back to ‘McIntosh’ (Fig. 2; Table S5). The increased-susceptibility haplotype of ‘Honeycrisp’, 6R, was inherited from ‘Duchess of Oldenburg’ (Fig. 2; Supplementary Table S5). ‘Minnewashta’ was deduced to have 6R, which was likely inherited from ‘State Fair’ but Chr. 6 QTL haplotype assignment in ‘State Fair’ was inconclusive (Fig. 2 and Supplementary Table S5). Examination of extended Chr. 6 haplotypes revealed that ‘Honeycrisp’ and ‘Minnewashta’ homologs matched for Chr. 6. ‘Sansa’s copy of 6R was traced to ‘Worcester Pearmain’ (Fig. 2 and Supplementary Table S5).

### Chromosome 7 QTL haplotype analysis

Twenty-one Chr. 7 QTL haplotypes, constructed from 26 SNPs spanning 6.7 cM (9.6-Mbp; Supplementary Table S4), were inherited from IBPs (Supplementary Table S7). Three haplotypes (7B, 7P, 7T) had significant effects in both 2016 and 2017 (*p* < 0.05; Fig. 1 and Supplementary Table S7). Three other haplotypes (7F, 7I, 7L) exhibited significant effects in 2017 (*p* < 0.05) but not 2016 (*p* > 0.05). The other 15 haplotypes did not have significant effects (*p* > 0.05) in either year. Generally, offspring that inherited 7P (*n* = 8) typically had low-susceptibility responses with an adjusted SLB BLUP mean of 0.19 across years (Fig. 1 and Supplementary Tables S7, S8). Offspring that inherited 7B (*n* = 38) or 7T (*n* = 7) demonstrated moderate to high susceptibility responses, with adjusted SLB BLUP means across years of 0.60 and 0.66, respectively (Fig. 1).

The reduced-susceptibility haplotype 7P of ‘Enterprise’ was inherited from Co-op 7 (Fig. 2 and Supplementary Table S7). The increased-susceptibility haplotype 7B of IBPs ‘Ginger Gold’, ‘Golden Delicious’, ‘Honeygold’, ‘Pinova’, ‘Silken’, and ‘Sunrise’ was traced back to ‘Grimes Golden’ (Fig. 2 and Supplementary Table S7). ‘Sunrise’ had a second copy of 7B that was likely inherited from PCF-3-120 (Fig. 2 and Supplementary Table S7). ‘Sansa’s increased-susceptibility haplotype 7T was traced back to ‘Worcester Pearmain’ (Fig. 2 and Supplementary Table S7).

### Chromosome 15 QTL haplotype analysis

Twelve IBP Chr. 15 QTL haplotypes, constructed from 17 SNPs spanning 6.1 cM (2.8 Mbp; Supplementary Table S4), segregated in full-sib families (Supplementary Table S9). Three haplotypes (15A, 15J, 15L) exhibited significant effects in both years (*p* < 0.05; Fig. 1). The other nine haplotypes did not have significant (*p* > 0.05) effects in either year. In general, offspring that inherited 15J (*n* = 20) or 15L (*n* = 12) demonstrated low to moderate susceptibility levels with adjusted SLB BLUP means across years of 0.35 and 0.20, respectively, while offspring that inherited 15A (*n* = 155) demonstrated moderate to high susceptibility levels with an adjusted SLB BLUP mean of 0.53 across years (Fig. 1 and Supplementary Tables S9, S10).

‘Enterprise’s reduced-susceptibility 15L haplotype was traced to its parent, Co-op 7 (Fig. 2 and Supplementary Table S9). The reduced-susceptibility haplotype 15J of ‘WA 5’ appeared to have resulted from recombination of Co-op 15’s 15D (no significant effect itself) with an uncharacterized haplotype (Fig. 2 and Supplementary Table S9). Nineteen IBPs (i.e., ‘Arlet’, ‘Aurora Golden Gala’, BC 8S-27-43, ‘Cripps Pink’, ‘Ginger Gold’, ‘Golden Delicious’, ‘Granny Smith’, ‘Honeygold’, ‘Minnewashta’, UMN selection 1839, NY 03, NY 06, ‘Pinova’, ‘Regent’, ‘Silken’, ‘Splendour’, ‘Sunrise’, ‘Sweet Sixteen’, and ‘WA 5’) had the increased-susceptibility 15A haplotype that was traced to multiple ancestors: ‘Frostbite’, ‘Grimes Golden’, ‘McIntosh’, ‘Rome Beauty’, and the unknown parent of ‘Fireside’ (Fig. 2 and Supplementary Table S9).

### Combined chromosomes 6, 7, and 15 QTL effects

**Fig. 3:**
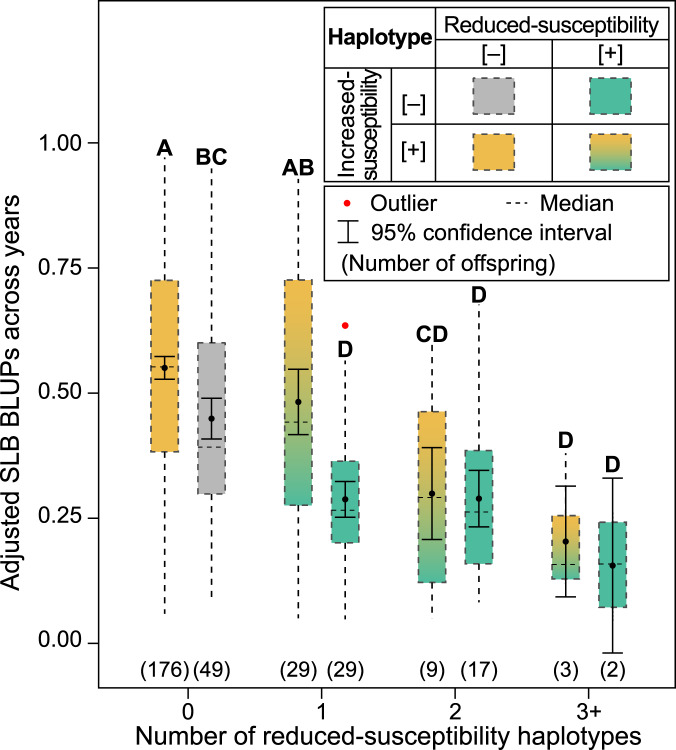
Higher numbers of reduced-susceptibility haplotypes across QTLs associated with lower susceptibility to fire blight. Distributions, means, and 95% confidence intervals of adjusted shoot length blight best linear unbiased predictions (SLB BLUPs) across years for 314 apple offspring with increased-susceptibility haplotypes present and absent grouped by a number of reduced-susceptibility haplotypes at a combination of three QTLs on chromosomes 6, 7, and 15. Offspring with haplotype 6R, 7B, 7T, and/or 15A were considered to have increased-susceptibility haplotypes present; reduced-susceptibility haplotypes were 6E, 6N, 6O, 7P, 15J, and 15L (corresponding with designations in Fig. 1). Offspring with neutral-effect haplotypes (i.e., haplotypes exhibiting non-significant effects in 2016 and/or 2017) at all three QTLs were considered to have no increased- and reduced-susceptibility haplotypes (gray bar). Different mean separation letters represent the least significant differences with a Bonferroni *p* adjustment (*α* < 0.05)

Average phenotypic variances explained (±standard deviations) by the Chrs. 6, 7, and 15 QTLs, and all QTLs combined, were 12 ± 3%, 9 ± 1%, 7 ± 1%, and 28 ± 2%, respectively. In general, the higher the number of reduced-susceptibility haplotypes offspring had across QTLs, the lower the adjusted SLB BLUPs (Fig. 3). The presence of increased-susceptibility haplotypes also affected offspring responses to fire blight (Fig. 3). In the presence of increased-susceptibility haplotypes, adjusted SLB BLUP means for offspring with zero, one, two, and ≥ 3 reduced-susceptibility haplotypes were 0.55, 0.48, 0.30, and 0.20, respectively, whereas in the absence of increased-susceptibility haplotypes adjusted SLB BLUP means for offspring with zero, one, two, and ≥3 reduced-susceptibility haplotypes were 0.45, 0.29, 0.29, and 0.16, respectively (Fig. 3). In the absence of increased-susceptibility haplotypes, similar responses were observed among offspring with one or more reduced-susceptibility haplotypes (Fig. 3). Offspring with two reduced-susceptibility haplotypes at Chr. 6 had similar responses to offspring with one reduced-susceptibility haplotype, and no offspring had two reduced-susceptibility haplotypes at the Chr. 7 QTL or Chr. 15 QTL (Supplementary Table S11). All offspring with ≥ 3 putative reduced-susceptibility haplotypes across QTLs (*n* = 5) were characterized by adjusted SLB BLUPs ≤ 0.38, with most having responses of ≤0.25 in both years (Supplementary Table S11). An offspring with four putative reduced-susceptibility haplotypes (6E, 6N, 7P, and 15L) had the lowest SLB BLUPs of both years (Supplementary Table S11). Forty-nine offspring were characterized by haplotypes exhibiting non-significant effects in 2016 and/or 2017 (i.e., neutral-effect haplotypes; Fig. 3).

### Frequency of chromosomes 6, 7, and 15 QTL haplotype alleles among 91 apple cultivars

Chr. 6, 7, and 15 QTL haplotypes were assigned via identity-by-descent (IBD) or identity-by-state (IBS) to 91 apple cultivars with previously reported^23^ evaluations for the degree of susceptibility to fire blight. Four, 21, and 17 of these cultivars had reduced-susceptibility haplotypes 6E, 6N, and 6O, respectively, six cultivars had reduced-susceptibility haplotype 7P, and four cultivars had reduced-susceptibility haplotype 15L (Table 2). In general, cultivars with one or more reduced-susceptibility haplotype at the Chrs. 6, 7, or 15 QTLs were classified as moderately susceptible (MS) to moderately resistant (MR; Table 2). Nine, seven, two, and 35 cultivars had increased-susceptibility haplotypes 6R, 7B, 7T, and 15A, respectively (Table 2). More than 60% of cultivars with one or more increased-susceptibility haplotype at the Chrs. 6, 7, or 15 QTLs were classified as HS to MR (Table 2).

**Table 2:** Haplotypes assigned by state within the chromosome 6, 7, and 15 resistance/susceptibility to fire blight QTLs for 91 apple cultivars

Colored haplotypes had significant effects (*p* < 0.05) among offspring in the pedigree-connected reference germplasm set in 2016 and 2017. Green colored haplotypes are putatively associated with reduced susceptibility whereas gold-colored haplotypes are putatively associated with increased susceptibility. Haplotypes denoted by italics were imputed

^a^Important breeding parent (IBP) or progenitor represented in the pedigree-connected germplasm set used for QTL analyses (Fig. 2)

^b^Range in resistance/susceptibility classifications (i.e., highly resistant [HR], moderately resistant [MR], intermediate [I], moderately susceptible [MS], highly susceptible [HS]) of cultivars evaluated by Kostick et al.^23^

^c^Chromosome 6 QTL haplotypes assigned by state. 6E, 6N, and 6O were consistently associated with reduced susceptibility whereas 6R was consistently associated with increased susceptibility (*p* < 0.05 in 2016 and 2017) among offspring in the pedigree-connected germplasm set

^d^Chromosome 7 QTL haplotypes assigned by state. 7P was consistently associated with reduced susceptibility whereas 7B and 7T were consistently associated with increased susceptibility (*p* < 0.05 in 2016 and 2017) among offspring in the pedigree-connected germplasm set

^e^Chromosome 15 QTL haplotypes assigned by state. 15J and 15L were consistently associated with reduced susceptibility whereas 15A was consistently associated with increased susceptibility (*p* < 0.05 in 2016 and 2017) among offspring in the pedigree-connected germplasm set

## Discussion

QTLs on Chrs. 6, 7, and 15 were consistently detected and characterized across pedigree-connected breeding families derived from 27 IBPs. A rare reduced-susceptibility allele at the Chr. 6 QTL was identified in ‘Honeycrisp’^40^, an IBP and economically important U.S. apple cultivar. The new genetic information obtained of estimated haplotype effects has potential utility in targeting reduced susceptibility to fire blight in apple breeding.

### Bi-allelic QTL model fit

FlexQTL^TM^ software applies a bi-allelic QTL model that assumes the presence of two functional alleles (*Q* and *q*) at a QTL, where *Q* is associated with high (e.g., high susceptibility) and *q* with low (e.g., low susceptibility) phenotypic values. In this study, *Q*/*q* allele assignments by FlexQTL^TM^ software for several IBPs were largely inconclusive likely due to the presence of multiple functional alleles with differing quantitative effects (Supplementary Table S3). A similar conclusion was made by Verma et al.^34^; two QTLs associated with fruit acidity in a pedigree-connected apple germplasm set were detected. Verma et al.^34^ reported that the presence of a third, intermediate-effect allele might have hindered accurate estimation of parental QTL genotypes at detected QTLs. The presence of multiple functional alleles in this study could explain the detection of other inconsistent QTLs (e.g., on Chr. 16). Under-representation of alleles might have also made it challenging for FlexQTL™ software to estimate some parental QTL genotypes. Lower observed fire blight severity levels in 2017 compared to 2016 in the dataset used here^7,23^ could have contributed to inconsistencies in *Q*/*q* allele assignments across years due to less distinct phenotypic contrasts between functional allele groups.

Resistance/susceptibility alleles were defined via analyses of SNP haplotypes within QTL intervals. Haplotypes that did not have consistently significant effects could be neutral-effect alleles or were under-represented. Because the approach used to determine effects of individual haplotypes (within-year one-way ANOVAs) examined haplotypes individually, small sample sizes and/or effects of haplotypes at other QTLs might have led to coincidental associations; however, it provided a framework for identifying haplotypes of interest.

### Identities of chromosomes 6, 7, and 15 QTLs

The Chr. 6 and Chr. 15 QTLs colocalized with previously reported QTLs, while the Chr. 7 QTL is possibly a novel locus. Based on colocalization on the GDDH13 reference genome sequence^21^, the Chr. 6 QTL detected in this study (Table 1) might be the same Chr. 6 QTL reported by Khan et al.^16^. The Chr. 15 QTL detected in this study colocalized with the QTL near SSR marker Hi04c05 (~24.4 Mbp) reported by Durel et al.^11^, Desnoues et al.^10^, and Khan et al.^16^ also reported QTLs on Chr. 15; lack of common markers made it difficult to determine whether the Chr. 15 QTL reported here colocalized with the Chr. 15 QTLs detected in those two studies^10,16^. In this study, the Chr. 7 QTL interval consistently detected was 22–32 cM (9.11–20.9 Mbp), which indicated it was distinct from the Chr. 7 QTLs of ‘Fiesta’^9,15^ and ‘Enterprise’^20^. In 2016 but not in 2017, there was positive evidence for a second Chr. 7 QTL at a similar position (lower part of Chr. 7; ~29–33-Mbp) to the previously reported Chr. 7 QTL of ‘Enterprise’^20^. The low representation of ‘Enterprise’ (*n* = 19 offspring) in this study might explain why a QTL on the lower portion of Chr. 7 was not consistently detected.

### Rare reduced-susceptibility haplotype at chromosome 6 QTL in ‘Honeycrisp’

This is the first report of a QTL associated with the degree of susceptibility to fire blight in ‘Honeycrisp’-derived families. FlexQTL^TM^ software’s estimate that ‘Honeycrisp’ was heterozygous for the Chr. 6 QTL supported the haplotype analysis findings. Found exclusively in ‘Honeycrisp’ and its progenitors, haplotype 6E was significantly associated with reduced susceptibility albeit of moderate effect compared with 7P and 15L (Fig. 1 and Supplementary Table S5). Haplotype 6E was traced to ‘Frostbite’, a cultivar previously classified as highly resistant (HR; Fig. 2)^23^. To our knowledge, haplotype 6E is a rare reduced-susceptibility allele and appears to be unique to germplasm derived from ‘Frostbite’ (Fig. 2). Previously reported MS to HR levels of cultivars Frostbite, Honeycrisp, Keepsake, and WA 38 might be partially explained by the presence of 6E (Table 2)^23^. In addition to inheriting 6E from ‘Honeycrisp’, ‘WA 38’^41^ inherited reduced-susceptibility haplotype 6N from ‘Enterprise’, which could explain ‘WA 38’s lower susceptibility classification compared to ‘Honeycrisp’^23^. The presence of increased-susceptibility haplotype 6R, which segregated in families derived from ‘Honeycrisp’, ‘Minnewashta’, and ‘Sansa’, might partially explain the previously reported HS to MS levels of several cultivars (Table 2). The extended haplotype sharing across the Chr. 6 QTL between ‘Honeycrisp’ and ‘Minnewashta’ indicated that haplotype 6R in these two cultivars possibly originated from a common unknown ancestor. Selection for haplotype 6E and against 6R might be a useful approach to developing breeding populations with lower susceptibility to fire blight.

### Novel putative fire blight QTL on chromosome 7

Significant Chr. 7 haplotypes (7B, 7P, 7T) had relatively low representation or their effects might have been confounded by haplotypes at other QTLs. Detection of the Chr. 7 QTL was likely due to segregation of the increased-susceptibility haplotype 7B in a ‘Pinova’ × NY 03 full-sib family with 22 offspring. Because reduced-susceptibility haplotype 7P was only present with reduced-susceptibility haplotypes at other QTLs (Supplementary Table S11), the association between haplotype 7P and reduced susceptibility might have been coincidental. Increased-susceptibility haplotype 7T should be considered putative because it was only represented by seven offspring. Conclusions about the effects of reduced-susceptibility haplotype 7P and increased-susceptibility haplotypes 7B and 7T were limited by small family sizes, variability between years for some haplotypes, and putative effects of haplotypes at other QTLs. Therefore, the Chr. 7 QTL should be considered putative and needs to be validated in future studies.

### Increased-susceptibility haplotype at chromosome 15 QTL prevalent among IBPs

Increased-susceptibility haplotype 15A was prevalent among IBPs and cultivars, which was likely due to the presence of ‘Golden Delicious’ in the pedigrees of many cultivars (Fig. 2). For example, haplotype 15A in 14 of 19 IBPs was inherited from ‘Grimes Golden’ through ‘Golden Delicious’ (Fig. 2 and Supplementary Table S9). The presence of haplotype 15A might partially explain HS to MS classifications of several cultivars (Table 2)^23^. The prevalence of haplotype 15A among IBPs indicates that it will likely segregate among offspring in many breeding families. Future selection against haplotype 15A may eliminate individuals with a higher potential to be susceptible.

### Highly resistant cultivar ‘Enterprise’ had four reduced-susceptibility haplotypes

The identification of four reduced-susceptibility haplotypes in ‘Enterprise’ was not surprising as ‘Enterprise’ has previously demonstrated strong levels of resistance to fire blight^20,23^. Because FlexQTL^TM^ software estimated that ‘Enterprise’ was homozygous *qq* at the three QTLs, it was expected that all Chrs. 6, 7, and 15 QTL haplotypes inherited from ‘Enterprise’ would be significantly associated with reduced susceptibility. However, SNP haplotype analyses within the QTL intervals revealed that four (6N, 6O, 7P, 15L) of Enterprise’s six haplotypes had significant effects, which indicated that ‘Enterprise’ was homozygous (*qq*) for the Chr. 6 QTL and heterozygous (*Qq*) for the Chr. 7 and Chr. 15 QTLs. Low representation (*n* = 19 offspring) of ‘Enterprise’s haplotypes might have hampered accurate estimation of haplotype effects. Therefore, reduced-susceptibility haplotypes traced to ‘Enterprise’ should be considered putative.

### Interactions at and among detected QTLs were not purely additive

Conclusions regarding combined QTL effects were limited by lack or under-representation of some genotype classes at and across QTLs. Under-representation of genotype classes is a common limitation of multi-locus studies in pedigree-connected germplasm. The approach used to examine combined QTL effects was like the *Q*-allele dosage model used by Verma et al.^34^. Although conclusions about the effects of specific compound QTL genotypes were limited, this approach increased statistical power by eliminating the need to examine specific allelic combinations separately.

Non-additive interactions among fire blight QTLs have been previously reported^20^ and were observed in this study. Under a purely additive model, adjusted SLB BLUP means were expected to be significantly lower with each additional reduced-susceptibility haplotype regardless of whether increased-susceptibility haplotypes were present or absent. Because additional reduced-susceptibility haplotypes did not always correspond to significantly lower susceptibility (Fig. 3), there might have been dominance effects at a QTL and/or epistatic interactions among QTLs. However, empirical examination of dominance effects and/or epistatic interactions was not possible in this study.

### Reduced-susceptibility haplotypes were present in several susceptible cultivars

The presence of putative reduced-susceptibility alleles in susceptible cultivars was not surprising and suggested epistatic interactions among multiple QTLs. Resistance alleles have been previously identified in susceptible individuals. For example, ‘Enterprise’s resistance allele at a major Chr. 7 QTL was traced by van de Weg et al.^20^ to its MS progenitor, ‘Cox’s Orange Pippin’. In this study, multiple HS to MS cultivars (e.g., Gala, Hudson, Jonathan, Melrose, Northern Spy, Yellow Newtown) had reduced-susceptibility alleles, which indicated that parental susceptibility classifications might not be predictive of offspring susceptibility levels due to segregation at multiple additives and/or epistatic QTLs. The development of trait-predictive DNA tests for detected QTLs might enable a more informed selection of parents for generating breeding populations with low susceptibility to fire blight.

### Functional annotations

Examination of functional annotations provided a list of annotated genes within the Chrs. 6, 7, and 15 QTL intervals, which could be used as a preliminary framework for a candidate gene approach. Further studies would be needed to identify and characterize the causal genes underlying detected QTL intervals.

### Detection and characterization of chromosome 8 QTL(s) were hampered

Multiple Chr. 8 QTLs might underlie variation in resistance/susceptibility to fire blight in apple, but the characterization of Chr. 8 QTL(s) detected in this study was limited. Because the location of Chr. 8 QTL(s) could not be precisely determined, Chr. 8 was not targeted for further analyses. Van de Weg et al.^20^ reported a putative, epistatic QTL at 30.6 cM (~10.1 Mbp) on Chr. 8. The Chr. 8 QTL interval of 22–40 cM (9.4–22.0 Mbp; Table 1) overlapped with the putative epistatic QTL detected by van de Weg et al.^20^. Detection and characterization of Chr. 8 QTL interval(s) could have been hampered by limited families examined, relatively small family sizes, and/or phenotyping method utilized.

### Study limitations

A limitation of this study was reliance on phenotypic data from inoculation with a single *E. amylovora* strain^7^. *E. amylovora* strains vary for virulence, which can result in variable fire blight incidence and severity^5,8,42,43^ and complicate elucidation of the genetic basis of the degree of susceptibility to fire blight. However, this study provided an experimental framework that can be replicated with different *E. amylovora* strains.

The large residual variation in the phenotypic dataset leveraged in this study was observed and managed^7^. Because several environmental, host, and pathogen factors impact fire blight incidence and severity^4–6^, large residual variation is common in fire blight field experiments. In this study’s germplasm set, Kostick et al.^7^ reported that 42–49% of the variation for SLB values was associated with the residual variation. The large experimental variation was managed by using adjusted SLB BLUPs as phenotypic values in QTL analyses.

Small family sizes likely limited the detection of QTLs, characterization of haplotypes, and examination of QTL × QTL interactions. Small family sizes combined with the uneven representation of some IBPs’ genomes, referred to as “skewed average allelic representation”^28^, led to under-representation or no representation of some haplotypes and compound QTL genotypes. Due to trait complexity, van de Weg et al.^20^ argued that the use of a single large family to dissect resistance/susceptibility to fire blight might be a more useful QTL analysis approach, although only for the few alleles able to segregate in a single family. Inevitably, the representation of some haplotypes was limited; however, haplotypes characterized in this study will enable targeted crosses to be made for future studies to validate haplotype effects. For example, an individual (e.g., ‘Keepsake’, ‘Frostbite’, ‘WA 38’) that is heterozygous for the rare reduced-susceptibility haplotype 6E could be used as a parent in a biparental QTL study.

### Breeding implications

The complexity of resistance to fire blight will continue to make breeding for resistance challenging. QTL haplotypes characterized in this study had moderate effects in the pedigree-connected reference germplasm set. A better understanding of alleles at detected QTLs and their interactions is needed before information gained can be used to develop and deploy trait-predictive DNA tests for the Chr. 6, Chr. 7, and Chr. 15 QTLs detected in this study.

Reduced- and increased-susceptibility allele information gained has utility in targeting reduced susceptibility to fire blight in breeding. In the short-term, allele information can be used to inform parental selection for more targeted crosses. Parents that have multiple increased-susceptibility and no reduced-susceptibility alleles at reported QTLs (e.g., ‘Ginger Gold’, ‘Minnewashta’, ‘Pinova’, ‘Sansa’, and ‘Sunrise’) should be avoided if strong resistance to fire blight is expected in the next generation. In contrast, cultivars homozygous for reduced-susceptibility alleles at QTLs and/or with reduced-susceptibility alleles at multiple QTLs (e.g., ‘Enterprise’) could be useful as parents when breeding for reduced-susceptibility to fire blight. DNA-informed parental selection combined with targeted phenotypic seedling selection in the greenhouse might be an effective approach to developing breeding populations with lower incidence and severity of fire blight. Additionally, the Chr. 6 QTL might be a useful target for DNA test development due to the breeding relevance of ‘Honeycrisp’, high QTL repeatability across years, and phenotypic variation explained by this QTL. Selection for haplotype 6E in ‘Honeycrisp’-derived parents (e.g., ‘WA 38’) or selection against offspring with haplotype 6R might be valuable approaches to developing breeding populations with low susceptibility to fire blight. Although reduced susceptibility is not complete resistance, a successful combination of multiple reduced-susceptibility alleles from different sources will contribute to achieving more durable resistance^8^. In the long-term, breeders should focus on selecting against increased-susceptibility alleles and pyramiding resistance alleles at major genes derived from wild germplasm with reduced-susceptibility alleles to achieve durable resistance to fire blight. Techniques like fast-track breeding^44–46^ could be used to accelerate introgression and pyramiding of favorable alleles.

## Materials and methods

### Germplasm

Germplasm in this study, a subset of the U.S. Apple Crop Reference and Breeding Pedigree Sets described by Peace et al.^28^, were represented in triplicate, propagated on M.111 rootstock, in a randomized complete block design field planting. The germplasm, planting establishment, and maintenance were previously described by Kostick et al.^7,23^. The final pedigree-connected germplasm set, described by Kostick et al.^7^, consisted of 32 F_1_ full-sib families with 314 unselected offspring representing 27 IBPs: ‘Arlet’, ‘Aurora Golden Gala’, BC 8S-27–43, ‘Braeburn’, ‘Cripps Pink’, ‘Delicious’, ‘Enterprise’, ‘Fuji’, ‘Ginger Gold’, ‘Golden Delicious’, ‘Granny Smith’, ‘Honeycrisp’, ‘Honeygold’, ‘Minnewashta’, MN 1964, NY 03, NY 06, ‘Pinova’, ‘Regent’, ‘Sansa’, ‘Silken’, ‘Splendour’, ‘Sunrise’, ‘Sweet Sixteen’, UMN selection 1839, ‘WA 5’, and a WSU apple breeding program selection, W.7 (Supplementary Table S12). ‘Honeycrisp’ was highly represented with 112 direct offspring. As described by Kostick et al.^7^, these 32 families were included in analyses due to sufficient average allelic representation (AAR) of their respective IBPs (≥ 12.5 AAR units)^28^.

### Phenotypic data

Phenotypic data used in this study were previously described^7^. In 2016 and 2017, up to 10 independent, actively growing shoots per tree (≤3 trees per individual) were inoculated with *E. amylovora* 153n^47,48^ in a replicated field inoculation study^7^. For each inoculated shoot, the response to fire blight was quantified as the proportion of the current season’s shoot length that was blighted (SLB). SLB values for each inoculated shoot were calculated by dividing the length of a necrotic lesion within the current season’s growth by the total shoot length. Statistical analyses of offspring SLB data were previously described^7^ and are briefly summarized here. Using R version 3.5.2 (R Core Team, Vienna, Austria), SLB data were analyzed across and within years with linear mixed models fit by restricted maximum likelihood (REML) via the lme4 package^49^. Within a year, blocks were considered fixed effects whereas offspring and block × offspring were considered random effects^7^. Normal QQ plots (i.e., sample vs. theoretical quantiles plots) were used to check the normality of random effects and residuals (Supplementary Figs. S5 and S6)^7^. Within a year, SLB best linear unbiased predictions (BLUPs), adjusted by trait means (as in Amyotte et al.^50^), were used to estimate offspring responses. Across year offspring SLB BLUPs were also estimated (data not shown). High adjusted SLB BLUPs (closer to 1.00) indicated high relative susceptibility to fire blight whereas low values (closer to 0.00) indicated low relative susceptibility. Adjusted SLB BLUPs were used as phenotypic values in QTL analyses and within-year SLB BLUP distributions are summarized here. In both 2016 and 2017, offspring responses spanned the spectrum ranging from highly resistant to highly susceptible (Supplementary Table S12). Adjusted SLB BLUPs ranged from 0.04 to 0.90 with a relatively normal distribution and an average of 0.48 (*n* = 312 offspring) in 2016 and from 0.08 to 0.97 with an average of 0.48 (*n* = 314 offspring) in 2017 (Supplementary Table S12). In 2017, offspring responses were slightly skewed towards lower adjusted SLB BLUPs than in 2016 but still followed a relatively normal distribution.

### Genotypic data

As part of the USDA-SCRI RosBREED project^51–53^, a genotypic data set of the IBPs, offspring, and available progenitors were obtained from the use of the International RosBREED SNP Consortium apple 8 K SNP array v1 ^35^ after marker calling, filtering, and curation of SNP data by Vanderzande et al.^36^. Genetic positions of the 3855 filtered SNPs were obtained from the map described by Vanderzande et al.^36^.

### QTL detection

FlexQTL^TM^ software (www.flexqtl.nl)^26,37–39^, streamlined with VisualFlexQTL^TM^ software, was used for QTL analyses. FlexQTL^TM^ enables the application of pedigree-based QTL analysis (PBA) via Markov Chain Monte Carlo (MCMC) simulation. MCMC convergence was reached with the chosen FlexQTL^TM^ software parameter settings (Supplementary Table S13): as described in Bink et al.^26^, reliable estimates of trait means, numbers of QTLs, and their variances were not considered achieved until lengths of effective chain size for each were at least 100—depending on the run, such convergence required Markov chain lengths of 250,000 or 300,000. An additive, bi-allelic QTL model (*Q*/q) was used where *Q* and *q* are associated with high and low phenotypic values, respectively. A dominance model was also initially tested. Separate QTL analyses were conducted using adjusted SLB BLUPs for each year. Phenotypic data were only included for unselected offspring (*n* = 312 in 2016 and 314 in 2017) to avoid any biases from previous breeding selection (as in van de Weg et al.^20^ and Verma et al.^34^). To ensure reproducibility, two replicate runs with different starting seed numbers were carried out for each year (as in van de Weg et al.^20^, Verma et al.^34^, and Howard et al.^32,33^).

QTL significance and stability were determined using the Bayes factor parameter (BF; 2lnBF_10_) and posterior intensity values. Evidence for a QTL was considered positive, strong, or decisive if BF values were > 2, 5, or 10, respectively^54^. Similar to van de Weg et al.^20^, QTL intervals were summarized in consecutive 2 cM “bins” (chromosomal segments) with BF > 2 and QTL interval boundaries were defined by the furthest left and right cM positions, respectively, of the two outer bins. The mode within a detected QTL interval was considered the most probable QTL position. The proportion of phenotypic variance explained by each significant QTL within a replicate run was estimated by dividing the variance explained by the total phenotypic variance (described by Verma et al.^34^). The proportions of phenotypic variance explained were only reported for repeatable QTLs. For purposes of describing QTL detection results, a single replicate run from each year is presented in the main text (as in Howard et al.^32^) and results of individual replicate runs are presented in Supplementary Tables S1 and S3.

### Functional annotation

Functional annotations of genes within each stable QTL interval were examined. Physical positions of stable QTL intervals using the GDDH13 v1.1 reference genome^21^ were used to determine the list of genes to consider. Available gene functional annotations for these intervals were obtained using JBrowse^55^ accessed via the Genome Database for Rosaceae (https://www.rosaceae.org)^56^. Genes with putative roles in disease resistance were particularly sought.

### QTL genotyping and haplotype characterization of QTL alleles

QTLs targeted for haplotype characterization had strong evidence (BF > 5) in at least one year and at least positive evidence (BF > 2) in the other year. FlexQTL^TM^ software was used to estimate QTL genotypes (*qq*, *Qq*, *QQ*), where *q* and *Q* were associated with low and high adjusted SLB BLUPs, respectively.

Haploblocks, i.e., chromosomal segments in which no recombination was observed among selected material (cultivars, selections) of the germplasm set, were previously defined by Vanderzande et al.^36^. Haploblocks used were chosen based on their proximity to detected QTL modes (peaks). SNP haplotypes of these haploblocks were constructed across the germplasm set using Pedihaplotyper software^57^ with SNP marker phasing via FlexQTL^TM^ software. Where necessary, deduction of haplotypes was conducted by examining available progenitor and/or progeny SNP haplotype data. Haplotypes were traced through the pedigree to the furthest ancestor and back through descendants via identity-by-descent (IBD). In contrast, shared haplotypes that could not be traced to a common ancestor/founder were considered identical-by-state (IBS). Where an ancestor lacked SNP information, haplotypes were deduced according to what it must have contributed to immediate offspring and assigned as homozygous for a haplotype where there was only a single deduced haplotype. Pedigrees were visualized using Pedimap software^58^.

Within-year one-way analysis of variances (ANOVAs; R version 3.5.2; R Core Team, Vienna, Austria) for each haplotype were performed to determine if adjusted SLB BLUPs were statistically different for presence vs. absence of a given haplotype. A haplotype with ANOVA *p* < 0.05 in 2016 and 2017 was marked as a significant-effect haplotype. Significantly higher and lower adjusted SLB BLUP means indicated increased- and reduced-susceptibility^8^ (i.e., relatively high and low susceptibility) haplotypes, respectively. Using R-package ‘ggpubr’^59^, adjusted SLB BLUP means, 95% confidence intervals, and distributions across years were determined and plotted for haplotypes with significant effects in both years.

To simultaneously examine the effects of multiple reduced-susceptibility haplotypes, offspring were first grouped by presence/absence of increased-susceptibility haplotypes and then by their number (i.e., 0, 1, 2, ≥ 3) of reduced-susceptibility haplotypes across detected QTLs. Adjusted SLB BLUP means, 95% confidence intervals, and distributions across years were determined and plotted for each of these eight groups of offspring. Statistical mean separation was calculated using the least significant difference with a Bonferroni *p* adjustment for multiple comparisons via R package ‘agricolae’^60^.

## Supplementary information

Figure S1

Figure S2

Figure S3

Figure S4

Figure S5

Figure S6

Combined Tables S1 - S13

## Supplementary information

**Supplementary Information** The online version contains supplementary material available at (10.1038/s41438-021-00466-6).
