# Supplementary material for: Fire blight QTL analysis in a multi-family apple population identifies a reduced-susceptibility allele in ‘Honeycrisp’
Source: Hortic Res. 2021 Feb 1;8:28. doi: 10.1038/s41438-021-00466-6 (PMC7847996; doi:10.1038/s41438-021-00466-6)

Figure S1. Replicate run 1 posterior intensity and sampling trace plots for QTL positions from FlexQTLTM software output for adjusted SLB BLUPs of 2016. Chromosome numbers are indicated at the top of each plot. Genetic coordinates (cM) indicate ends and middle of chromosomes.

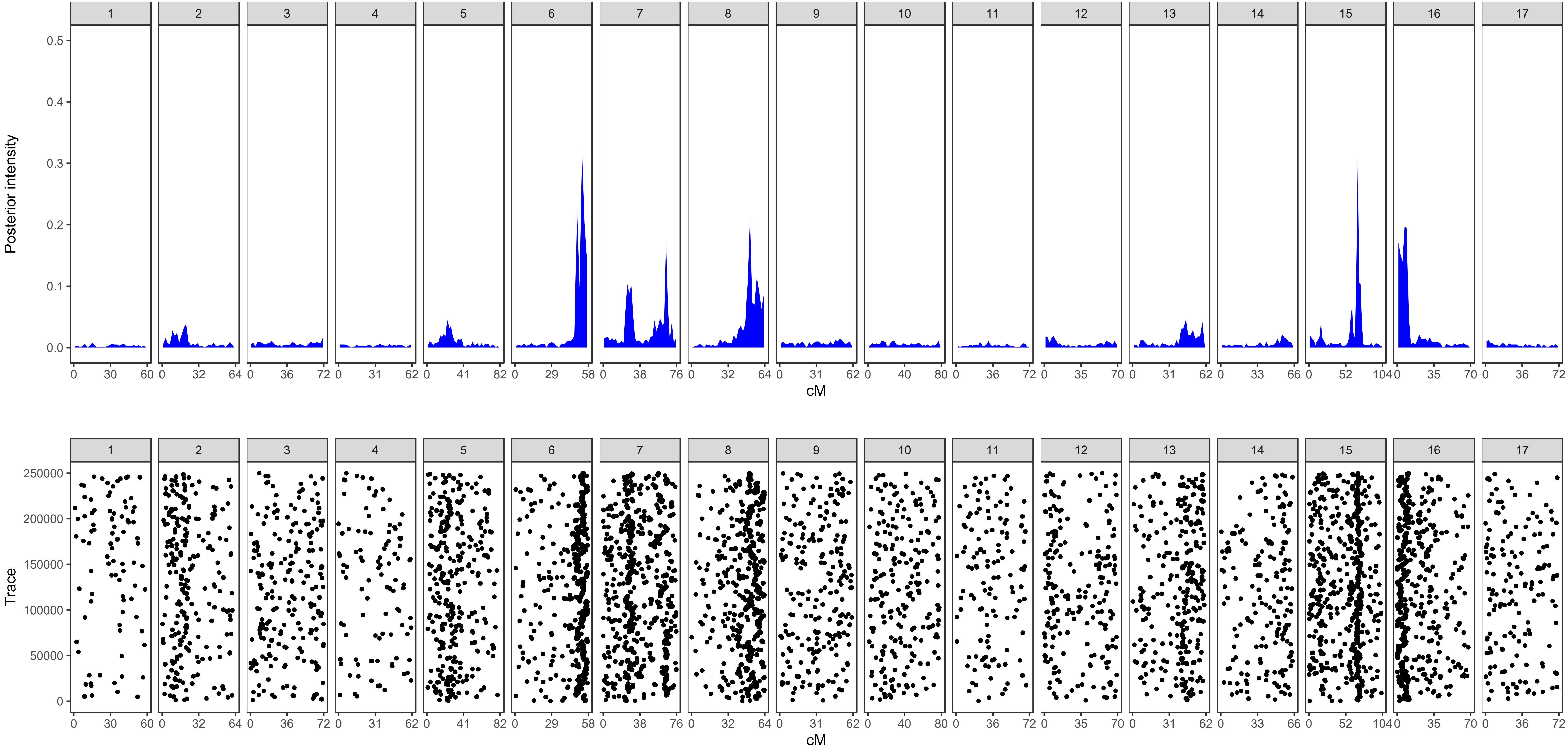

Supplement: Supplementary file 1 — Figure S1 [file 41438_2021_466_MOESM1_ESM.pdf]
