# Supplementary material for: Fire blight QTL analysis in a multi-family apple population identifies a reduced-susceptibility allele in ‘Honeycrisp’
Source: Hortic Res. 2021 Feb 1;8:28. doi: 10.1038/s41438-021-00466-6 (PMC7847996; doi:10.1038/s41438-021-00466-6)

Figure S3. Replicate run 1 posterior intensity and sampling trace plots for QTL positions from FlexQTL™ software output for adjusted SLB BLUPs of 2017. Chromosome numbers are indicated at the top of each plot. Genetic coordinates (cM) indicate ends and middle of chromosomes.

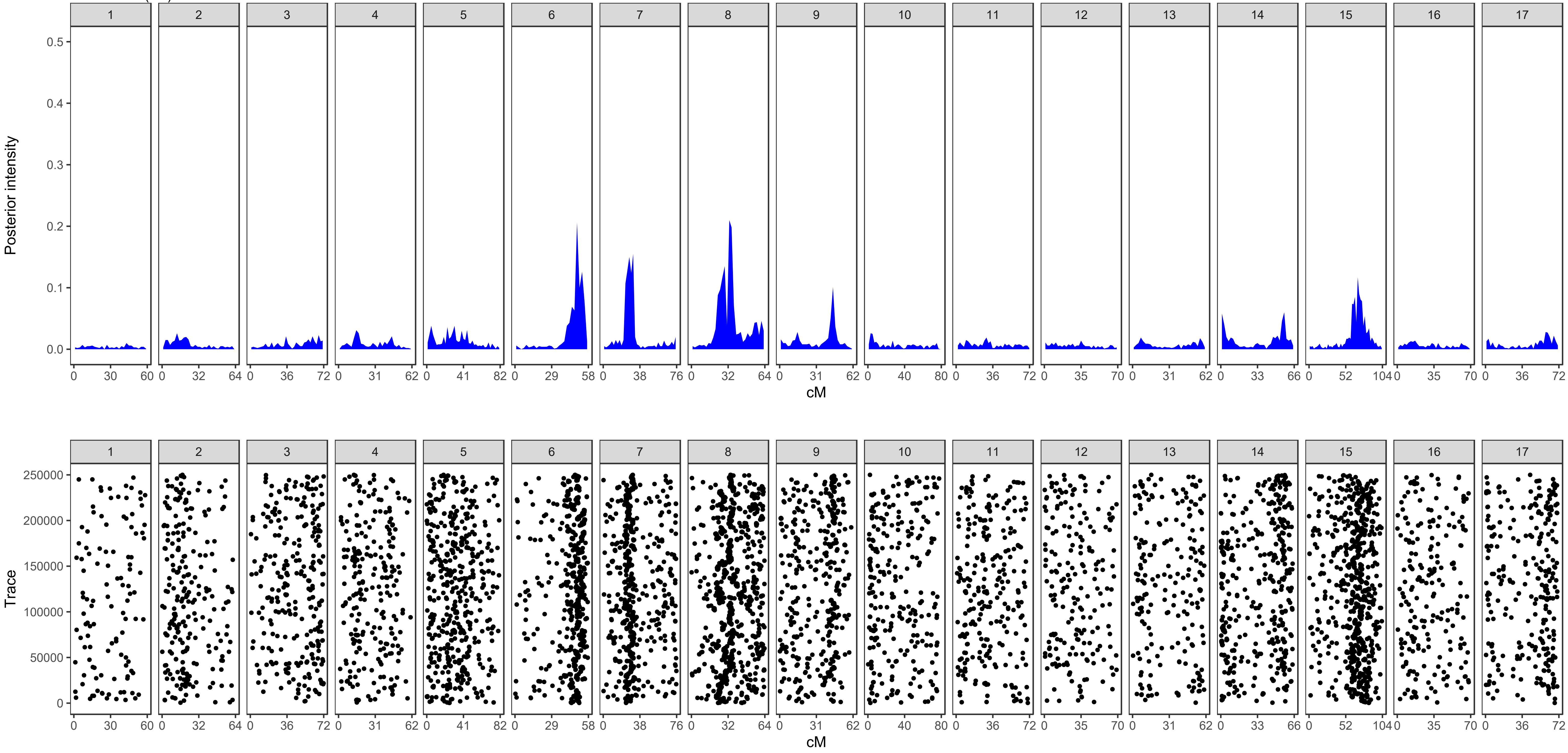

Supplement: Supplementary file 3 — Figure S3 [file 41438_2021_466_MOESM3_ESM.pdf]
