# Supplementary material for: Fire blight QTL analysis in a multi-family apple population identifies a reduced-susceptibility allele in ‘Honeycrisp’
Source: Hortic Res. 2021 Feb 1;8:28. doi: 10.1038/s41438-021-00466-6 (PMC7847996; doi:10.1038/s41438-021-00466-6)
Supplement: Supplementary file 5 — Figure S5 [file 41438_2021_466_MOESM5_ESM.pdf]

Figure S5. Normal QQ (i.e., sample versus theoretical quantiles) plots used to check normality of random effects (i.e., block × offspring interaction, offspring) and residuals of the mixed linear model fit to 2016 shoot length blighted (SLB) data described by Kostick *et al.*[1]

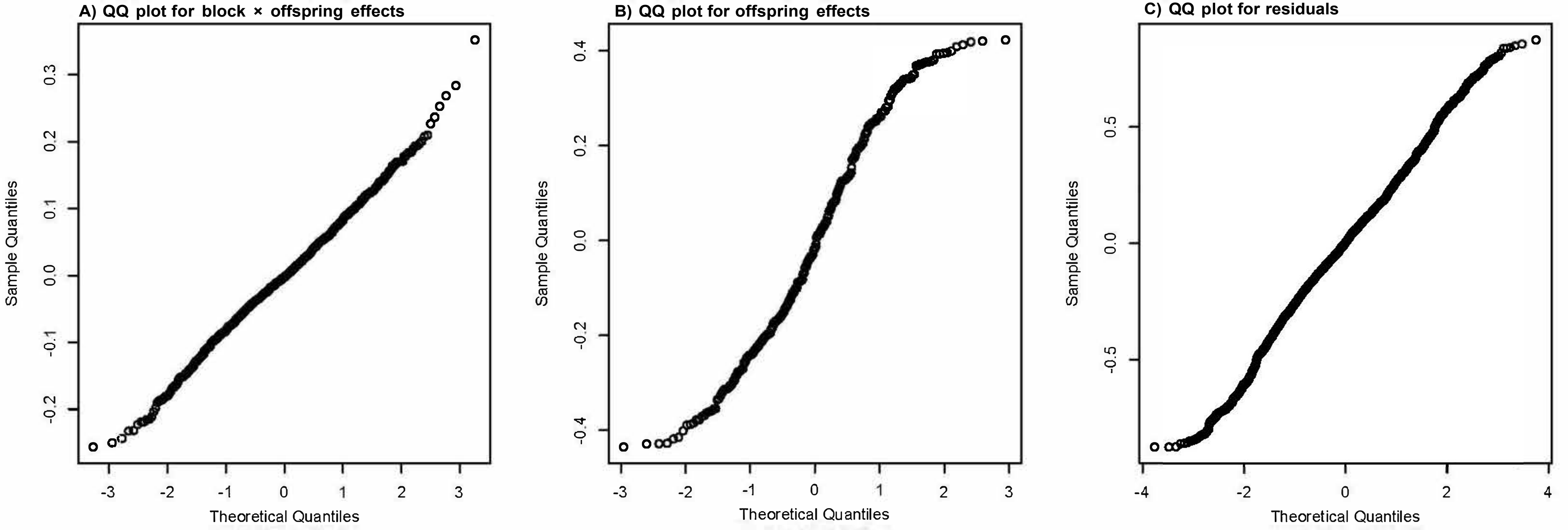

[1]Kostick, S.A., Norelli, J.L., Teh, S. & Evans, K.M. Quantitative variation and heritability estimates of fire blight resistance in a pedigree-connected apple germplasm set. *J. Plant Pathol.* (2020). https://doi.org/10.1007/s42161-020-00543-0
